# Supplementary material for: LiBiNorm: an htseq-count analogue with improved normalisation of Smart-seq2 data and library preparation diagnostics
Source: PeerJ. 2019 Feb 4;7:e6222. doi: 10.7717/peerj.6222 (PMC6366399; doi:10.7717/peerj.6222)
Supplement: Supplemental Information 1 [file peerj-07-6222-s001.pdf]

LiBiNorm: An HTSeq analogue with improved normalisation of Smart-seq2 data and library preparation diagnostics

Nigel P. Dyer, Vahid Shahrezaei, Daniel Hebenstreit

Supplementary Data

1. Review of other bias removal methods

Several methods and software releases have been published which attempt to cater for different sources of bias that can occur when assessing expression levels using RNA-seq data. None of these consider the global bias that is corrected for using LiBiNorm but instead they mostly assume an overall linear scaling between read numbers and RNA lengths and focus on local deviations from uniform coverage.

One form of such local bias is associated with the relationship between DNA sequence and the likelihood of reads at any given position within a transcript. Cufflinks and Callisto include an algorithm that compensates for this bias [1], as does mSeq [2]. Similarly, Alpine [3] corrects for systematic errors due to GC bias. Mix^2 [4] uses global bias correction in order to improve the quantification of isoform expression and differential gene expression. It makes no association between the bias and the parameters of the associated protocol so is not able to correct for errors in absolute expression levels that are introduced by such global bias.

Biases can also be an issue when quantifying isoform expression. Approaches for reducing the effects of such bias include RSEM [5] and the algorithm developed by BE Howard and S Heber [6] which make use of the read distributions within genes with multiple isoforms. rQuant [7, 8] uses a similar approach but does not allow for length dependent read distributions. The same is true for the algorithm proposed by W Li and T Jiang [9], Multisplice[10], and PennSeq [11]. The approach described by Z Wu, X Wang and X Zhang [12] for improving isoform quantification uses a single length independent bias in the distribution of reads in a gene, and so does not correct for the effect of global bias on relative expression of genes of different length. L Wan, X Yan, T Chen and F Sun [13] considers one specific contributing mechanism to global bias, that of RNA degradation, and use a single exponential distribution model again to improve isoform quantification.

There are a small number of bias corrections that do recognise that there may be a length related global bias in RNA-seq data. Sailfish [14, 15] and its successor Salmon [16], include a single length parameter in its multi-bias correction model, assuming a simple linear length associated bias. This therefore fails to recognise the multi-parameter complexity of the global bias described by N Archer, MD Walsh, V Shahrezaei and D Hebenstreit [17]. The flux simulator [18] recognises the significance of global bias but only allows the effects of this to be simulated and does not provide a method for inferring parameters from a dataset or correcting for the bias in the dataset, aside from some shortcomings of its bias models [17]. Maxcounts [19] recognises the errors introduced by global bias but then uses the maximum number of overlapping reads in a transcript as a bias-independent means of measuring expression levels. The resulting values are thus a function of a very small proportion of the reads, increasing the uncertainty associated with the expression levels and making them vulnerable to local sequence related bias.

2. Bias models

Bias correction in LiBiNorm is based on fitting functions to the coverage by sequencing reads along transcripts. Different functions are available that correspond to different models of how the coverages arise. The functions/models in general depend on transcript length and describe how the coverage shapes change with length, which we call the ‘global bias’ (in contrast to local coverage variation within transcripts).

This global bias is mainly introduced by cDNA conversion, which can be done in different ways and which is the main feature that discriminates different experimental protocols for RNA-seq library preparation. Our various models are designed to cater to those differences. Note that the main target of

LiBiNorm is bias correction for Smart-seq2 datasets, requiring model BD (see below). The others are included for completeness and can be useful for testing purposes.

Common variations of cDNA production include the starting points of reverse transcription/1st strand synthesis (e.g. at the 3' ends of transcripts by primers targeting the poly-A tail or internally by random priming etc) or 2nd strand synthesis (e.g. at the end of 1st strands by poly-A tagging etc). A central feature of our models is the description of the cDNA conversion as a stochastic process, where synthesis endpoints are probability distributions that depend on the enzymes' processivities.

Our different models predict characteristic overall shapes of the length dependent coverages, but specific aspects, such as the slopes of the coverage curves, are subject to parameters. It is the estimation of these parameters through fitting of our models that allows the global bias to be corrected; this is because the two main parameters, t_1 and t_2, correspond to the 1st and 2nd strand processivities, a major determinant for the varying effectiveness to produce cDNA along transcripts, thus introducing global bias.

A summary of the models LiBiNorm includes, and the coverage functions, library preparation characteristics and typical coverage curves associated with these, are shown below. Derivation of the models and more detailed descriptions can be found in our previous work [17]. Models A and C are included for completeness only and are based on unrealistic assumptions (perfect enzymatic conversions, i.e. infinite processivities), while model E applies to random-priming based datasets, which is hardly in use anymore. Most relevant is model BD, which builds on the models B and D (see below).

For simplicity, we did not include here the additional parameters of d and h, which describe a reduced coverage (by factor d+1) at the ends of transcripts (over h bases) due to reduced fragmentation efficiency. l corresponds to the transcript length, x is the position within the transcript, t_1 and t_2 correspond to the 1st and 2nd strand processivities, respectively. a corresponds to the proportion of PCR enrichment of full-length 2nd strands for SMART protocols. Processivity parameters for model E (t_1', t_2') have slightly different interpretations and include further scaling factors α_1 and α_2, please see [17] for details.

**Model**	**Coverage function (excluding *d*, *h*)**<br>$f(x, l, \ldots) =$	**Characteristics of cDNA conversion**	**Typical library preparation protocol**	**Expected coverage along ~3kb mRNA**<br>**5' 3'**
**A**	$e^{-l(t_1+t_2)}$	Terminally primed 1st and 2nd strands; both full-length conversion	None. (infinite enzyme processivities)	
**B**	$\frac{1}{(t_1+t_2)}[t_1 e^{(x-l)(t_1+t_2)} + t_2 e^{-l(t_1+t_2)}]$	Terminally primed 1st and 2nd strands; partial 1st strand conversion, full-length 2nd strand	None. (infinite 2nd strand processivity)	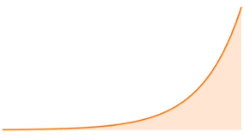
**C**	$e^{-t_1 l - t_2 x}$	Terminally primed 1st and 2nd strands; full-length 1st strand, partial 2nd strand conversion	None. (infinite 1st strand processivity)	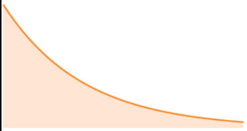

**D**	$\frac{1}{(t_1+t_2)}[t_1 e^{-t_1(l-x)} + t_2 e^{-t_1 l - t_2 x}]$	Terminally primed 1st and 2nd strands; both partial conversion	Poly-A tagging	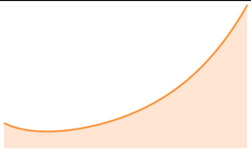
**BD**	$a\,\mathbf{B} + (1-\ a)\,\mathbf{D}$	Combination of B and D (PCR-based enrichment of full-length 2nd strands)	Smart-seq	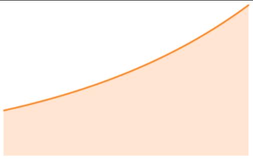
**E**	$\frac{\alpha_1\alpha_2}{t_1'(t_1'+t_2')}[l - \frac{1}{t_1'+t_2'} - \frac{1}{t_1'} - \frac{t_1' e^{-l(t_1'+t_2')}}{t_2'(t_1'+t_2')} + \frac{(t_1'+t_2')\, e^{-lt_1'}}{t_1' t_2'}]$	Internally primed 1st and 2nd strands (random primers and RNaseH nicking, respectively); both partial conversion	Random priming	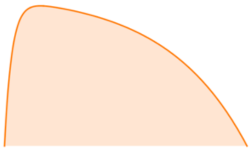

3. Evaluation of bias Removal - Methods

The evaluation of bias removal was done using the *Drosophila* RNA-seq data generated using the Smart-seq2 and TruSeq protocols as part of an investigation into low cost RNA-seq protocols [20]. As part of this study, the authors introduced up to 20% *D. virilise* RNA in the *D. Melanogaster* RNA as a natural RNA spike-in in order to assess expression quantification accuracy. They also performed the Smart-seq2 protocol with 2.5-fold and 5-fold dilution of the Nextera reagents as well as at the recommended concentration in order to gauge potential cost savings. These dilutions provide variation in the associated conditions for both the TruSeq and the Smart-seq2 protocol and allow the global bias normalisation to be tested across this range of conditions.

A simple R^2 measure of correlation was used to evaluate the reduction in global bias associated with Smart-seq2 data using LiBiNorm and this same measure was used to evaluate the performance of other expression quantification packages which contain some degree of bias removal.

The reference expression levels were the expression levels in Transcripts per Kilobase Million (TPM) [5] for the four TruSeq samples (SRR1743167 to SRR1743170). The R^2 statistic was then used to quantify the correlation between the $\log_2$ of these reference expression levels and those of the 14 Smart-seq2 samples (SRR1743153 to SRR1743166). There will be a number of factors that result in the correlation being less than perfect. As well as the biological and technical noise associated with such measurements, the effect of global bias, which is particularly pronounced in the Smart-seq data, will decrease the correlation. Any reduction in the global bias upon correction efforts will improve the correlation, which would be seen as an increase in the R^2 statistic towards 1.0, indicating a perfect correlation.

These R^2 statistics were calculated before and after LiBiNorm was used to reduce the global bias present in the Smart-seq2 data. The improvement was expressed as a percentage where an R^2 value of 1.0 would be a 100% improvement using the following formula:

$$I(\%) = {}^{1}\!/_{100} \frac{R^2 - R^2_{ref}}{1 - R^2_{ref}},$$

where $I(\%)$ is the percentage improvement, R^2_{ref} is the R^2 value for the reference linear TPM expression levels and R^2 is the R^2 value with the global bias reduced Smart-seq2 data.

We repeated this with four other expression quantification packages; Cufflinks [1], Salmon [16], Mix^2 [4] and MaxCount [19]. In the case of Cufflinks and Salmon, the performance was assessed with and without the optional additional bias compensation that is available within the packages.

In all cases, the same gene definitions were used as defined in Drosophila_melanogaster. BDGP6.91.gtf, release 91 of the Ensemble *Drosophila melanogaster* gene annotation, which is based on the BDGP6 reference genome [21]. The details of how the gene annotation information is used is dependent on the software package being assessed.

When the RNA-seq data is analysed by LiBiNorm, the <fileroot>_counts.txt file produced by LiBiNorm shows the length and the read count for each gene and this is used to calculate the standard TPM expression values for each gene. Only those genes where the read counts for both the TruSeq and the Smart-seq2 data was greater than nine were used to quantify the correlation between the two sets of expression values.

The quantification of correlation values for the other packages for each Smart-seq2/TruSeq combination was restricted to the same set of genes in order help ensure the comparisons were as equivalent as possible.

LiBiNorm

Read alignment and expression quantification were performed as described in the main body of the paper.

The <filename>_counts.txt provides the bias corrected TPM values as well as the transcript lengths and raw counts for each gene. An excel spreadsheet was used to calculate the standard linear TPM values, find the $\log_2$ expression values, calculate the R^2 value for those genes where the count exceeded 9 for both samples and plot the correlation graph.

Cufflinks

The following was used to evaluate the gene expression using Cufflinks:

```
cufflinks -p 4 -g Drosophila_melanogaster.BDGP6.91.gtf -G
Drosophila_melanogaster.BDGP6.91.gtf bamfiles/<fileroot>.bam
```

The –G option ensures that Cufflinks aligns and counts the reads just to the same transcript definitions as was used for the LiBiNorm evaluation.

Bias correction in Cufflinks is optional; the following was used to determine gene expression with this feature enabled:

```
cufflinks -b ../Drosophila_melanogaster.BDGP6.dna.toplevel.fa -p 4 -G
Drosophila_melanogaster.BDGP6.91.gtf <fileroot>.bam
```

The FKPM column from the genes.fpkm_tracking file was used to calculate the correlation between the TruSeq and Smart-seq2 results. The correlation is identical to that which would be found using TPM values because FPKM and TPM values for any RNA-seq dataset differ only by a single scaling factor.

Salmon

Salmon is not designed to work with reads that are aligned to a full genome but instead works with reads that have been aligned to a reference transcript set.

LiBiNorm includes a feature to create a fasta file that corresponds to the transcripts that it is using to determine gene expression. Such a fasta file, corresponding to the transcripts used for the LiBiNorm evaluation, was generated using:

```
LiBiNorm refSeq -i gene_id Drosophila_melanogaster.BDGP6.91.gtf
bdgp6_tran\genome_tran > refseq.fa
```

where `bdgp6_tran\genome_tran` is the root filename of the HISAT2 reference genome previously used to align the RNA-seq data.

Salmon was then used to convert the fasta file into a suitable index using:

```
salmon index -t refseq.fa -i index
```

Gene expression was then quantified using:

```
salmon quant -p 2 -i index -l A -r <fileroot>.fastq.gz -o <fileroot>_quant
```

Bias correction in Salmon is also optional and expression levels were generated with the feature enabled using:

```
salmon quant --seqBias --posBias -p 2 -i index -l A -r <fileroot>.fastq.gz -o
<fileroot>_quant
```

We calculated the R^2 statistic using the TPM values in the quant.sf files that were generated.

Mix^2

The same aligned reads used for the LiBiNorm analysis were used to evaluate Mix^2. Gene expression was then quantified using:

```
mix-square -o . -G Drosophila_melanogaster.BDGP6.91.gtf -B <fileroot>.bam
```

We calculated the R^2 statistic using the FPKM_CHN column of the genes_summary_<fileroot>.dat files.

MaxCount

The same bamfiles of aligned reads were used as for the LiBiNorm analysis.

The bedtools patch that implements the MaxCount algorithm was downloaded from:

http://sysbiobig.dei.unipd.it/?q=MAXCOUNTS&sid=2036

and applied to the bedtools2-2.19.1 source from the Cygwin command line. It was then compiled using gcc version 6.4.0 after having added the –fpermissive option as required within the makefile to ensure that the compilation ran to completion.

LiBiNorm includes a feature where it can create a bed file that corresponds to the transcripts that it is using to determine gene expression. Such a bed file, corresponding to the transcripts used for the LiBiNorm evaluation, was generated using:

```
LiBiNorm bed -i gene_id Drosophila_melanogaster.BDGP6.91.gtf > genome.bed
```

The count of the maximum number of overlapping reads within each exon was then calculated using

```
bedtools coverage -max -abam <fileroot>.bam -b genome.bed > <fileroot>_counts.txt
```

This produces a text file listing the maximum count for each exon in the bed file. An Excel pivot table was then used to find the maximum count across all of the exons associated with a specific gene.

4. Evaluation of bias removal – results

The reference for the analysis of bias removal was the R^2 correlation statistic of the comparison of the $\log_2$ of the linear TPM expression values for all 56 combinations of the Smart-seq2 and TruSeq data (Supplementary Figure 1 and Table 1).

These were then compared with the R^2 correlation statistics for the same 56 combinations when LiBiNorm was used to correct for the global bias in the Smart-seq2 data (Supplementary Figure 2 and Table 2). For each of these combinations the improvement in R^2 was calculated as a percentage and the mean improvement used as a measure of the effectiveness of the bias correction.

For comparison, a similar process was performed with Cufflinks, with and without the '-b' bias correction option (Supplementary Figures 3 & 4 and Tables 3 & 4), Salmon, with and without the '--seqBias --posBias' bias correction options (Supplementary Figures 5 & 6 and Tables 5 & 6), Mix[2] (Supplementary Figure 7 and Table 7) and MaxCount (Supplementary Figure 8 and Table 8).

In all of the following tables the sample identifiers are abbreviated for clarity, such that SRR1743153 is identified as ..153.

The following colour scales are used for all of the tables:

0.7000
0.7500
0.8000
0.8500
0.9000
0.9500

R^2 values

-30%
-20%
-10%
0%
10%
20%
30%

R^2 change with respect to reference

Reference Linear TPM comparison

Smart-seq2	Model	TruSeq 0% *D.v.* ..167	5% *D.v.* ..168	10% *D.v.* ..169	20% *D.v.* ..170
0% *D.v.*	..153	0.7048	0.6874	0.6998	0.7012
5% *D.v.*	..154	0.8310	0.8191	0.8238	0.8200
10% *D.v.*	..155	0.8036	0.7901	0.8238	0.7931
20% *D.v.*	..156	0.8224	0.8080	0.8155	0.8119
2.5-fold dilution		..167	..168	..169	..170
0% *D.v.*	..157	0.8921	0.8804	0.8878	0.8853
1% *D.v.*	..158	0.8904	0.8733	0.8854	0.8823
5% *D.v.*	..159	0.8646	0.8491	0.8580	0.8530
10% *D.v.*	..160	0.8663	0.8476	0.8607	0.8586
20% *D.v.*	..161	0.8311	0.8126	0.8221	0.8176
5-fold dilution		..167	..168	..169	..170
0% *D.v.*	..162	0.8916	0.8769	0.8862	0.8839
1% *D.v.*	..163	0.8870	0.8685	0.8816	0.8779
5% *D.v.*	..164	0.8609	0.8444	0.8537	0.8496
10% *D.v.*	..165	0.8580	0.8425	0.8546	0.8530
20% *D.v.*	..166	0.8398	0.8199	0.8305	0.8256

Supplementary Table 1. Reference R^2 correlations for the linear TPM expression values for all of the TruSeq/Smart-seq2 protocol combinations with different Nextera reagent dilutions and amounts of additional *D. virilise* RNA.

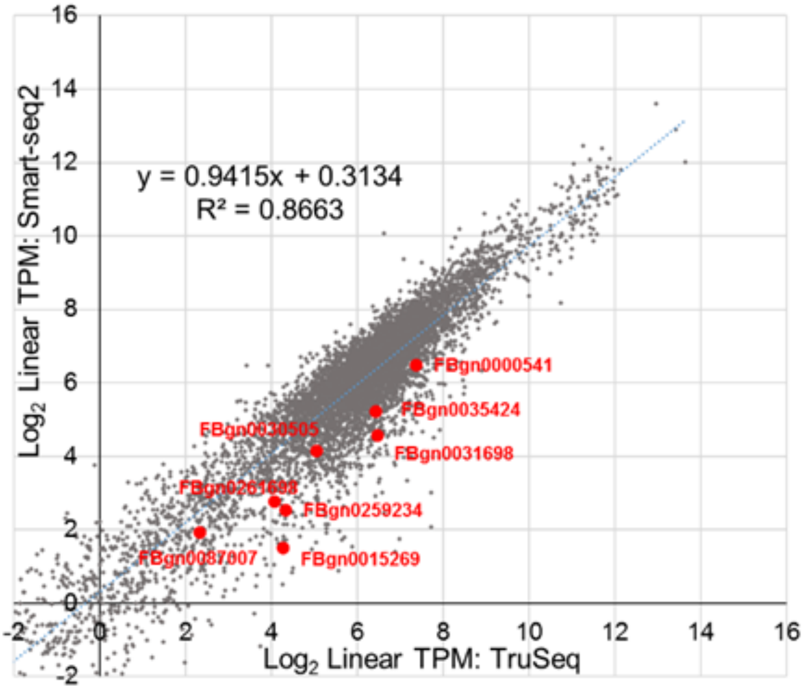

Supplementary Figure 1 (Figure 2a in the main paper). Correlation of the reference linear TPM gene expression for the SRR1743160/ SRR1743167 combination that is identified with a box in the table above.

Smart-seq2 bias correction using LiBiNorm

Smart-seq2	TruSeq		R^2		R^2 improvement			
	..167	..168	..169	..170	..167	..168	..169	..170
..153	0.7905	0.7823	0.7835	0.7833	29.0%	30.4%	27.9%	27.5%
..154	0.8525	0.8440	0.8457	0.8427	12.7%	13.7%	12.4%	12.6%
..155	0.8210	0.8092	0.8145	0.8123	8.9%	9.1%	-5.2%	9.3%
..156	0.8325	0.8195	0.8261	0.8238	5.7%	5.9%	5.8%	6.3%
	..167	..168	..169	..170	..167	..168	..169	..170
..157	0.9213	0.9162	0.9174	0.9157	27.0%	29.9%	26.4%	26.6%
..158	0.9242	0.9155	0.9195	0.9165	30.8%	33.3%	29.8%	29.1%
..159	0.8840	0.8735	0.8772	0.8716	14.3%	16.2%	13.5%	12.6%
..160	0.9089	0.9000	0.9029	0.9007	31.8%	34.3%	30.3%	29.8%
..161	0.8402	0.8298	0.8305	0.8247	5.4%	9.2%	4.7%	3.9%
	..167	..168	..169	..170	..167	..168	..169	..170
..162	0.9245	0.9176	0.9192	0.9173	30.3%	33.1%	29.0%	28.8%
..163	0.9210	0.9108	0.9157	0.9118	30.1%	32.2%	28.8%	27.8%
..164	0.8813	0.8709	0.8732	0.8681	14.7%	17.0%	13.3%	12.3%
..165	0.9024	0.8953	0.8995	0.8973	31.3%	33.5%	30.9%	30.2%
..166	0.8558	0.8429	0.8459	0.8406	10.0%	12.8%	9.1%	8.6%

Supplementary Table 2. R^2 correlation coefficients for the same combinations as was evaluated with the reference linear TPM analysis (Supplementary Table 1), except that the global bias corrected Smart-seq2 expression values are used (left). Percentage improvements in correlation compared to the reference comparison are also shown (right).

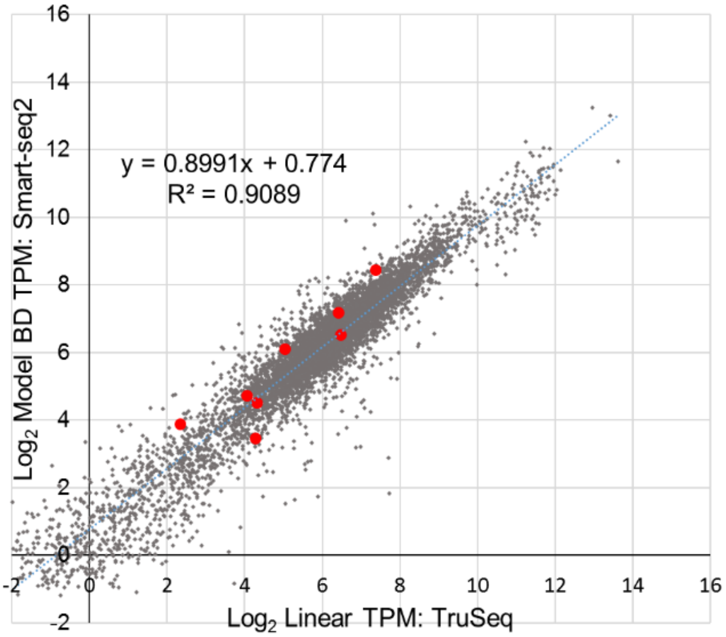

Supplementary Figure 2 (Figure 2b in the main paper). Correlation for the same SRR1743160/SRR1743167 combination as Supplementary Figure 1 with global bias correction applied to Smart-seq2 data.

Expression Quantification using Cufflinks

	TruSeq		R^2				R^2 improvement	
Smart-seq2	..167	..168	..169	..170	..167	..168	..169	..170
..153	0.7231	0.7062	0.7186	0.7201	6.2%	6.0%	6.3%	6.3%
..154	0.8366	0.8249	0.8296	0.8262	3.3%	3.2%	3.3%	3.5%
..155	0.8117	0.7984	0.8046	0.8019	4.1%	3.9%	-10.9%	4.2%
..156	0.8304	0.8168	0.8240	0.8210	4.5%	4.6%	4.6%	4.8%
	..167	..168	..169	..170	..167	..168	..169	..170
..157	0.8914	0.8799	0.8864	0.8845	-0.7%	-0.5%	-1.2%	-0.7%
..158	0.8879	0.8723	0.8829	0.8805	-2.3%	-0.8%	-2.3%	-1.5%
..159	0.8615	0.8468	0.8545	0.8504	-2.3%	-1.5%	-2.5%	-1.8%
..160	0.8631	0.8471	0.8576	0.8570	-2.4%	-0.4%	-2.2%	-1.2%
..161	0.8268	0.8093	0.8186	0.8157	-2.5%	-1.8%	-2.0%	-1.0%
	..167	..168	..169	..170	..167	..168	..169	..170
..162	0.8899	0.8759	0.8841	0.8829	-1.6%	-0.8%	-1.8%	-0.8%
..163	0.8844	0.8673	0.8790	0.8761	-2.3%	-0.9%	-2.1%	-1.5%
..164	0.8574	0.8421	0.8502	0.8469	-2.5%	-1.5%	-2.3%	-1.8%
..165	0.8557	0.8418	0.8522	0.8509	-1.6%	-0.4%	-1.7%	-1.4%
..166	0.8342	0.8142	0.8255	0.8216	-3.5%	-3.1%	-3.0%	-2.3%

Supplementary Table 3. As Supplementary Table 2 but with expression quantified using Cufflinks.

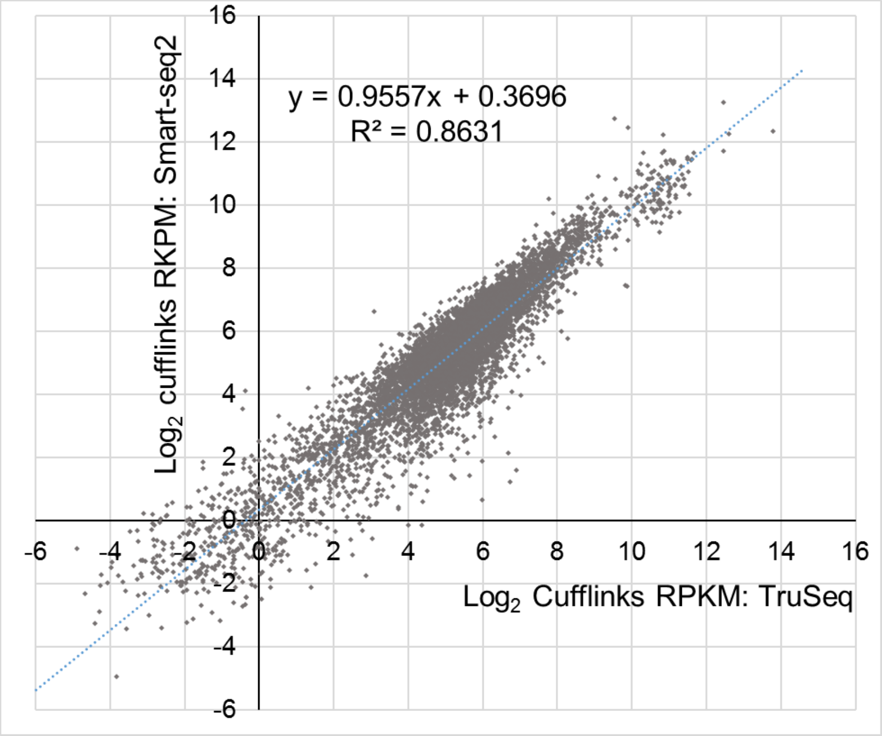

Supplementary Figure 3. Correlation of the same SRR1743160/SRR1743167 as Supplementary Figure 1 with expression quantified using Cufflinks.

Cufflinks with Bias Correction

Smart-seq2	TruSeq ..167	..168	R^2 ..169	..170		R^2 improvement ..167	..168	..169	..170
..153	0.6602	0.6424	0.6613	0.6656		-15.1%	-14.4%	-12.8%	-11.9%
..154	0.7882	0.7764	0.7889	0.7896		-25.4%	-23.6%	-19.8%	-16.9%
..155	0.7521	0.7378	0.7524	0.7527		-26.2%	-24.9%	-40.5%	-19.5%
..156	0.7824	0.7687	0.7833	0.7840		-22.5%	-20.5%	-17.5%	-14.8%

	..167	..168	..169	..170		..167	..168	..169	..170
..157	0.8659	0.8540	0.8643	0.8642		-24.3%	-22.1%	-20.9%	-18.4%
..158	0.8655	0.8480	0.8631	0.8621		-22.7%	-20.0%	-19.5%	-17.1%
..159	0.8263	0.8100	0.8244	0.8217		-28.3%	-25.9%	-23.7%	-21.3%
..160	0.8435	0.8265	0.8413	0.8414		-17.1%	-13.9%	-13.9%	-12.2%
..161	0.7867	0.7665	0.7832	0.7814		-26.3%	-24.6%	-21.8%	-19.8%

	..167	..168	..169	..170		..167	..168	..169	..170
..162	0.8721	0.8573	0.8700	0.8702		-18.0%	-15.8%	-14.2%	-11.7%
..163	0.8603	0.8412	0.8576	0.8564		-23.7%	-20.7%	-20.2%	-17.6%
..164	0.8214	0.8036	0.8188	0.8167		-28.3%	-26.2%	-23.8%	-21.9%
..165	0.8329	0.8204	0.8326	0.8339		-17.6%	-14.1%	-15.2%	-13.0%
..166	0.8036	0.7821	0.7992	0.7964		-22.6%	-21.0%	-18.4%	-16.8%

Supplementary Table 4. As Supplementary Table 3 but with the –b bias correction applied when the expression was quantified using Cufflinks.

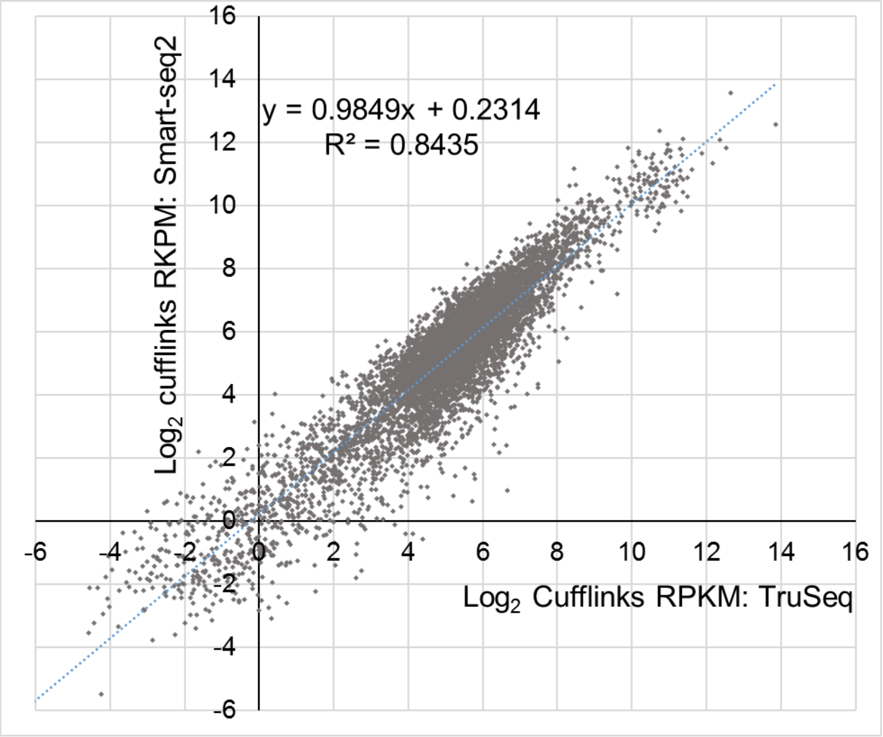

Supplementary Figure 4. Correlation of gene expression as Supplementary Figure 3 but with the –b bias correction applied when the expression was quantified using Cufflinks.

Salmon

Smart-seq2	TruSeq ..167	..168	R^2 ..169	..170	R^2 improvement ..167	..168	..169	..170
..153	0.7255	0.7077	0.7213	0.7221	7.0%	6.5%	7.2%	7.0%
..154	0.8361	0.8240	0.8296	0.8265	3.0%	2.7%	3.3%	3.6%
..155	0.8116	0.7975	0.8051	0.8016	4.1%	3.5%	-10.6%	4.1%
..156	0.8307	0.8167	0.8246	0.8215	4.7%	4.5%	4.9%	5.1%
	..167	..168	..169	..170	..167	..168	..169	..170
..157	0.8889	0.8801	0.8876	0.8855	-3.0%	-0.3%	-0.2%	0.2%
..158	0.8856	0.8711	0.8831	0.8810	-4.3%	-1.7%	-2.1%	-1.1%
..159	0.8612	0.8477	0.8552	0.8519	-2.5%	-0.9%	-2.0%	-0.7%
..160	0.8608	0.8473	0.8589	0.8583	-4.1%	-0.2%	-1.2%	-0.3%
..161	0.8247	0.8085	0.8184	0.8153	-3.8%	-2.2%	-2.1%	-1.3%
	..167	..168	..169	..170	..167	..168	..169	..170
..162	0.8874	0.8762	0.8849	0.8839	-3.9%	-0.5%	-1.1%	0.1%
..163	0.8817	0.8665	0.8791	0.8763	-4.7%	-1.5%	-2.1%	-1.3%
..164	0.8554	0.8426	0.8510	0.8484	-3.9%	-1.1%	-1.8%	-0.8%
..165	0.8558	0.8428	0.8534	0.8526	-1.5%	0.2%	-0.9%	-0.2%
..166	0.8327	0.8154	0.8265	0.8232	-4.4%	-2.5%	-2.3%	-1.4%

Supplementary Table 5. As Supplementary Table 2 but with expression quantified using Salmon.

Supplementary Figure 5. Gene expression correlation for the same SRR1743160/SRR1743167 combination as Supplementary Figure 1 but with expression quantified using Salmon.

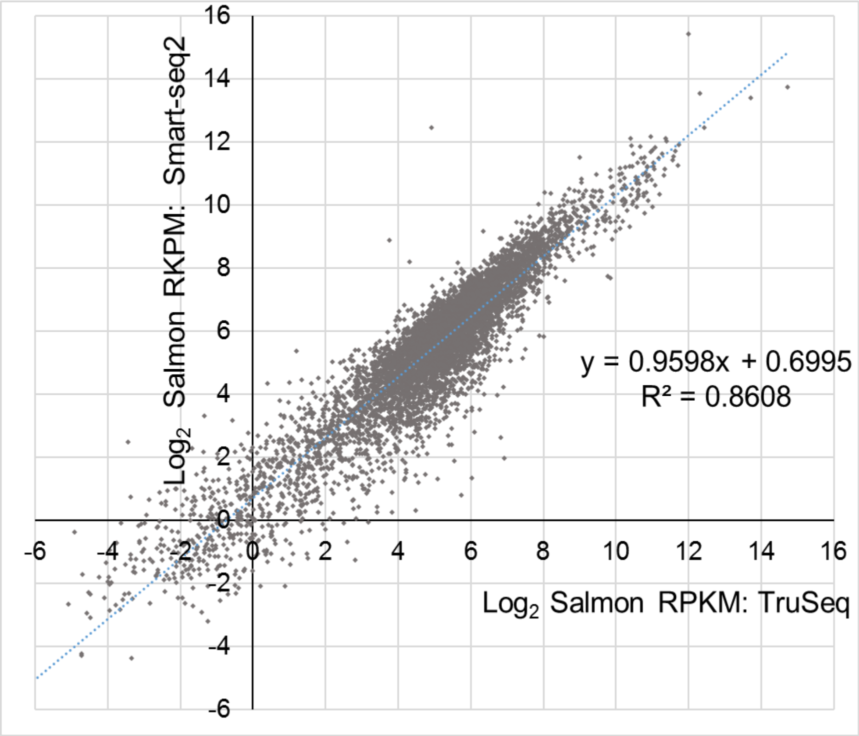

Salmon with Bias correction

Smart-seq2	TruSeq ..167	..168	R^2 ..169	..170	R^2 improvement ..167	..168	..169	..170
..153	0.7434	0.7279	0.7456	0.7494	13.1%	13.0%	15.3%	16.1%
..154	0.8176	0.8061	0.8181	0.8170	-8.0%	-7.2%	-3.2%	-1.7%
..155	0.7891	0.7757	0.7895	0.7877	-7.4%	-6.9%	-19.4%	-2.6%
..156	0.8133	0.8004	0.8141	0.8130	-5.1%	-4.0%	-0.7%	0.6%
	..167	..168	..169	..170	..167	..168	..169	..170
..157	0.8672	0.8585	0.8695	0.8684	-23.1%	-18.3%	-16.3%	-14.7%
..158	0.8633	0.8594	0.8748	0.8739	-24.7%	-11.0%	-9.3%	-7.1%
..159	0.8477	0.8343	0.8467	0.8441	-12.5%	-9.8%	-8.0%	-6.1%
..160	0.8544	0.8427	0.8574	0.8586	-8.9%	-3.2%	-2.3%	0.0%
..161	0.8143	0.7987	0.8130	0.8110	-9.9%	-7.4%	-5.1%	-3.6%
	..167	..168	..169	..170	..167	..168	..169	..170
..162	0.8748	0.8632	0.8756	0.8753	-15.6%	-11.1%	-9.3%	-7.4%
..163	0.8714	0.8565	0.8725	0.8710	-13.8%	-9.1%	-7.6%	-5.7%
..164	0.8444	0.8318	0.8452	0.8434	-11.8%	-8.0%	-5.8%	-4.1%
..165	0.8478	0.8364	0.8495	0.8508	-7.2%	-3.9%	-3.5%	-1.5%
..166	0.8212	0.8043	0.8195	0.8167	-11.6%	-8.7%	-6.5%	-5.1%

Supplementary Table 6. As Supplementary Table 5 but with --seqBias --posBias bias correction options applied when processing the data using Salmon.

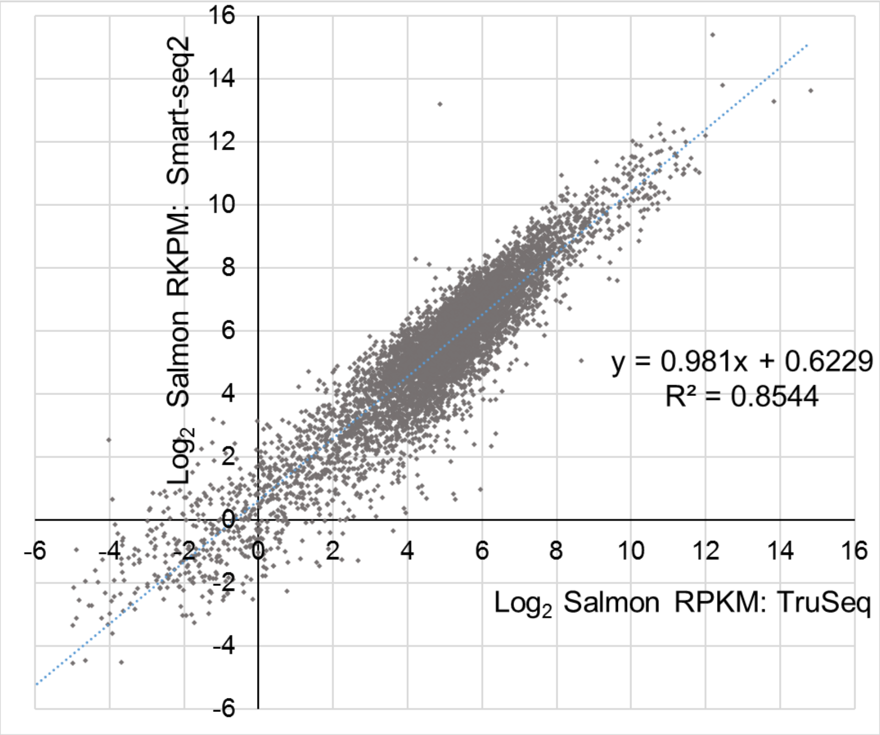

Supplementary Figure 6. Gene expression correlation as Supplementary Figure 5 but with the --seqBias --posBias bias correction options applied when processing the data using Salmon.

Mix^2

Smart-seq2	TruSeq ..167	..168	R^2 ..169	..170	R^2 improvement ..167	..168	..169	..170
..153	0.7002	0.6807	0.6952	0.6955	-1.5%	-2.2%	-1.5%	-1.9%
..154	0.8264	0.8140	0.8189	0.8149	-2.7%	-2.8%	-2.8%	-2.8%
..155	0.7982	0.7836	0.7907	0.7868	-2.8%	-3.1%	-18.8%	-3.1%
..156	0.8193	0.8048	0.8125	0.8089	-1.7%	-1.7%	-1.6%	-1.6%
	..167	..168	..169	..170	..167	..168	..169	..170
..157	0.8862	0.8722	0.8802	0.8779	-5.5%	-6.9%	-6.8%	-6.4%
..158	0.8809	0.8625	0.8748	0.8722	-8.7%	-8.5%	-9.3%	-8.5%
..159	0.8550	0.8374	0.8471	0.8425	-7.1%	-7.7%	-7.7%	-7.2%
..160	0.8550	0.8354	0.8491	0.8470	-8.5%	-8.0%	-8.3%	-8.2%
..161	0.8167	0.7964	0.8063	0.8030	-8.5%	-8.6%	-8.9%	-8.0%
	..167	..168	..169	..170	..167	..168	..169	..170
..162	0.8850	0.8682	0.8783	0.8756	-6.1%	-7.0%	-7.0%	-7.1%
..163	0.8767	0.8568	0.8699	0.8668	-9.1%	-8.9%	-9.9%	-9.1%
..164	0.8505	0.8320	0.8427	0.8379	-7.4%	-7.9%	-7.5%	-7.8%
..165	0.8477	0.8306	0.8435	0.8413	-7.2%	-7.6%	-7.7%	-7.9%
..166	0.8254	0.8026	0.8145	0.8106	-9.0%	-9.6%	-9.4%	-8.6%

Supplementary Table 7. As Supplementary Table 2 but with expression quantified using Mix^2.

Supplementary Figure 7. Gene expression correlation for the same SRR1743160/SRR1743167 combination as Supplementary Figure 1 but with expression quantified using Mix^2.

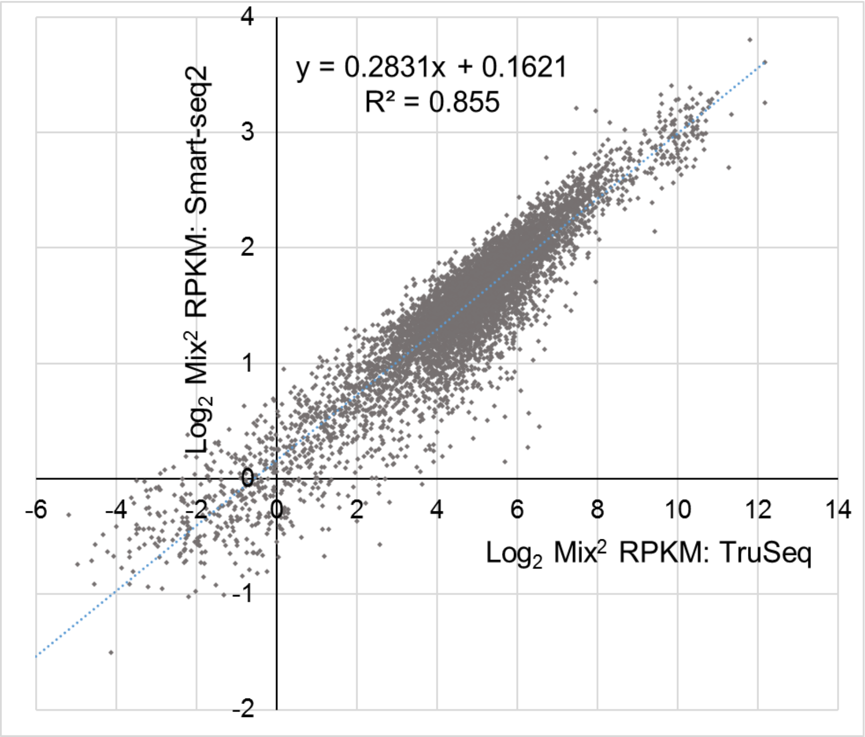

MaxCount

	TruSeq		R^2		R^2 improvement			
Smart-seq2	..167	..168	..169	..170	..167	..168	..169	..170
..153	0.7160	0.7079	0.7181	0.7150	3.8%	6.5%	6.1%	4.6%
..154	0.7677	0.7642	0.7651	0.7614	-37.5%	-30.4%	-33.3%	-32.5%
..155	0.7477	0.7415	0.7458	0.7408	-28.5%	-23.2%	-44.2%	-25.3%
..156	0.7808	0.7740	0.7791	0.7749	-23.4%	-17.7%	-19.7%	-19.7%

	..167	..168	..169	..170	..167	..168	..169	..170
..157	0.8453	0.8312	0.8392	0.8348	-43.4%	-41.2%	-43.3%	-43.9%
..158	0.8440	0.8272	0.8380	0.8323	-42.3%	-36.4%	-41.4%	-42.4%
..159	0.7923	0.7746	0.7870	0.7777	-53.4%	-49.4%	-50.0%	-51.2%
..160	0.8196	0.8031	0.8151	0.8111	-35.0%	-29.2%	-32.7%	-33.7%
..161	0.7610	0.7427	0.7494	0.7436	-41.5%	-37.3%	-40.9%	-40.6%

	..167	..168	..169	..170	..167	..168	..169	..170
..162	0.8475	0.8314	0.8406	0.8357	-40.7%	-36.9%	-40.1%	-41.5%
..163	0.8308	0.8129	0.8231	0.8178	-49.7%	-42.3%	-49.3%	-49.3%
..164	0.7952	0.7778	0.7880	0.7798	-47.2%	-42.8%	-44.9%	-46.4%
..165	0.8146	0.7990	0.8135	0.8079	-30.5%	-27.6%	-28.3%	-30.7%
..166	0.7763	0.7569	0.7673	0.7604	-39.6%	-34.9%	-37.2%	-37.4%

Supplementary Table 8. As Supplementary Table 2 but with expression quantified using MaxCount.

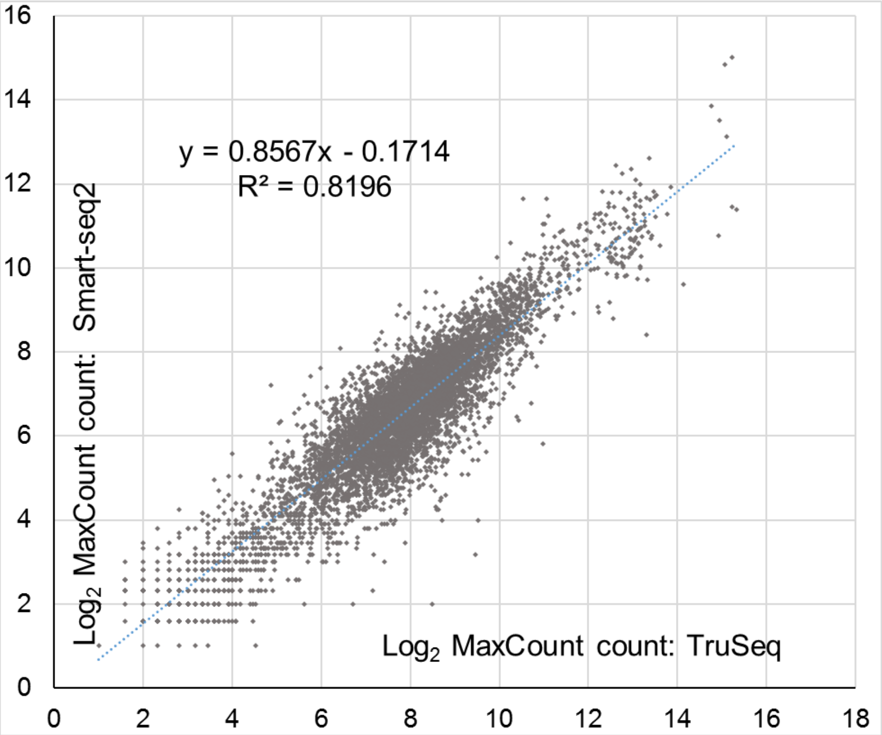

Supplementary Figure 8. Gene expression correlation for the same SRR1743160/SRR1743167 combination as Supplementary Figure 1 but with expression quantified using MaxCount.

1. Roberts A, Trapnell C, Donaghey J, Rinn JL, Pachter L: **Improving RNA-Seq expression estimates by correcting for fragment bias**. *Genome biology* 2011, **12**(3):R22.
2. Li J, Jiang H, Wong WH: **Modeling non-uniformity in short-read rates in RNA-Seq data**. *Genome biology* 2010, **11**(5):R50.
3. Love MI, Hogenesch JB, Irizarry RA: **Modeling of RNA-seq fragment sequence bias reduces systematic errors in transcript abundance estimation**. *Nature biotechnology* 2016, **34**(12):1287-1291.
4. Tuerk A, Wiktorin G, Güler S: **Mixture models reveal multiple positional bias types in RNA-Seq data and lead to accurate transcript concentration estimates**. *PLoS computational biology* 2017, **13**(5):e1005515.
5. Li B, Ruotti V, Stewart RM, Thomson JA, Dewey CN: **RNA-Seq gene expression estimation with read mapping uncertainty**. *Bioinformatics (Oxford, England)* 2010, **26**(4):493-500.
6. Howard BE, Heber S: **Towards reliable isoform quantification using RNA-SEQ data**. *BMC bioinformatics* 2010, **11 Suppl 3**:S6.
7. Bohnert R, Ratsch G: **rQuant.web: a tool for RNA-Seq-based transcript quantitation**. *Nucleic acids research* 2010, **38**(Web Server issue):W348-351.
8. Bohnert R, Behr J, Rätsch G: **Transcript quantification with RNA-Seq data**. *BMC bioinformatics* 2009, **10**(13):P5.
9. Li W, Jiang T: **Transcriptome assembly and isoform expression level estimation from biased RNA-Seq reads**. *Bioinformatics (Oxford, England)* 2012, **28**(22):2914-2921.
10. Huang Y, Hu Y, Jones CD, MacLeod JN, Chiang DY, Liu Y, Prins JF, Liu J: **A robust method for transcript quantification with RNA-seq data**. *Journal of computational biology : a journal of computational molecular cell biology* 2013, **20**(3):167-187.
11. Hu Y, Liu Y, Mao X, Jia C, Ferguson JF, Xue C, Reilly MP, Li H, Li M: **PennSeq: accurate isoform-specific gene expression quantification in RNA-Seq by modeling non-uniform read distribution**. *Nucleic acids research* 2014, **42**(3):e20.
12. Wu Z, Wang X, Zhang X: **Using non-uniform read distribution models to improve isoform expression inference in RNA-Seq**. *Bioinformatics (Oxford, England)* 2011, **27**(4):502-508.
13. Wan L, Yan X, Chen T, Sun F: **Modeling RNA degradation for RNA-Seq with applications**. *Biostatistics* 2012, **13**(4):734-747.
14. Patro R, Mount SM, Kingsford C: **Sailfish enables alignment-free isoform quantification from RNA-seq reads using lightweight algorithms**. *Nature biotechnology* 2014, **32**:462.
15. Zheng W, Chung LM, Zhao H: **Bias detection and correction in RNA-Sequencing data**. *BMC bioinformatics* 2011, **12**(1):290.
16. Patro R, Duggal G, Love MI, Irizarry RA, Kingsford C: **Salmon provides fast and bias-aware quantification of transcript expression**. *Nature methods* 2017, **14**(4):417.
17. Archer N, Walsh MD, Shahrezaei V, Hebenstreit D: **Modeling Enzyme Processivity Reveals that RNA-Seq Libraries Are Biased in Characteristic and Correctable Ways**. *Cell Syst* 2016, **3**(5):467-479 e412.
18. Griebel T, Zacher B, Ribeca P, Raineri E, Lacroix V, Guigo R, Sammeth M: **Modelling and simulating generic RNA-Seq experiments with the flux simulator**. *Nucleic acids research* 2012, **40**(20):10073-10083.
19. Finotello F, Lavezzo E, Bianco L, Barzon L, Mazzon P, Fontana P, Toppo S, Di Camillo B: **Reducing bias in RNA sequencing data: a novel approach to compute counts**. *BMC bioinformatics* 2014, **15 Suppl 1**:S7.
20. Combs PA, Eisen MB: **Low-cost, low-input RNA-seq protocols perform nearly as well as high-input protocols**. *PeerJ* 2015, **3**:e869.
21. Zerbino DR, Achuthan P, Akanni W, Amode M R, Barrell D, Bhai J, Billis K, Cummins C, Gall A, Girón CG *et al*: **Ensembl 2018**. *Nucleic acids research* 2018, **46**(D1):D754-D761.
